# Quantitative analysis, pharmacokinetics and metabolomics study for the comprehensive characterization of the salt-processing mechanism of Psoraleae Fructus

**DOI:** 10.1038/s41598-018-36908-w

**Published:** 2019-01-24

**Authors:** Kai Li, Ning Zhou, Xiao-Ke Zheng, Wei-Sheng Feng, Fei Li, Zhen-Ling Zhang, Ya-Qi Lu

**Affiliations:** 1 College of Pharmacy, Henan University of Chinese Medicine, Zhengzhou, 450046 China; 2 Collaborative Innovation Center for Respiratory Disease Diagnosis and Treatment & Chinese Medicine Development of Henan Province, Zhengzhou, 450046 China; 3 State Key Laboratory of Natural Medicines, China Pharmaceutical University, Nanjing, 210009 China

## Abstract

Research based on quantitative analysis, pharmacokinetics and metabolomics was conducted to explore the effects of salt-processing on Psoraleae Fructus (PF). Quantitative analysis showed that the contents of bioactive components were higher in salt-processed Psoraleae Fructus (SPF) extract than in PF extract. Pharmacokinetics indicated that the overall *AUC* and *t*_max_ levels was higher, while *C*_max_ was lower in the SPF group. In the metabolomics study, the differential influences of PF and SPF on 22 common biomarkers and associated metabolic pathways showed that salt-processing could enhance the effect of PF and reduce toxicity in the cardiovascular and renal systems. The internal correlations among these results, together with the influence of salt-processing, suggested that the effects of heating and newly generated surfactants during the salt-processing procedure were the primary causes of the changes in chemical composition and absorption characteristics, as well as the subsequent enhanced efficacy and minor toxicity.

## Introduction

*Psoralea corylifolia* L., a member of the Leguminosae family, is widely distributed in China, India, Burma and Sri Lanka^1^. Psoraleae Fructus (PF), the dried ripe fruit of *Psoralea corylifolia* L., is frequently used in prescriptions of traditional Chinese medicine (TCM) and health food due to its functions of preventing and/or treating various physical dysfunctions and diseases, e.g., osteoporosis^2^, bone fracture^3^ diarrhoea and asthma^4^. A variety of compounds have been isolated from PF, including coumarins^5^, flavonoids^6^ and monoterpene phenols^7^. Salt-processed Psoraleae Fructus (SPF), the most commonly used PF product in the clinic, exhibits stronger efficiency and minor toxicity in the renal system than PF.

The herb-processing method, which is based on herb characteristics and medical need, has been assisting TCM in developing reasonable curative effects for a long time. Processing can enhance the efficiency, reduce the toxicity and/or alter the original actions of TCM. The most widely used ways to process herbs include stir-frying with salt-water or wine, mix-frying with oil, stir-baking with bran, steaming with water or rice wine, and braising with liquorice liquids or rice wine. The change in chemical composition of the herbs before and after processing was considered to be the fundamental effect underlying herb-processing^8^.

Over the past decades, most research about processing-methods only focused on alterations in chemical composition^9,10^ or curative effects^11^ individually. However, the relationship between chemicals and efficacy, as well as absorption characteristics that are essential to the therapeutic effect of oral administration, have received little attention. To date, pharmacokinetics has been proven to be an efficacious approach to exploring the intracorporal course of drugs, especially absorption characteristics^12^. Metabolomics, a systems biology approach, is characterized by a holistic perspective consistent with the integral principle of TCM. The system-based mode has been successfully applied to evaluate the comprehensive efficacy of TCM^13^.

In light of the above, a novel strategy based on quantitative analysis, pharmacokinetics and metabolomics was proposed in this study to explore the internal correlations among chemical composition, absorption characteristics and comprehensive efficacy, as well as the influence of salt-processing. First, quantitative analysis of bioactive components in PF and SPF extracts was carried out to ascertain the alteration in chemical composition. Second, a pharmacokinetics study was conducted to explore the absorption characteristics of bioactive components. Lastly, a metabolomics study was performed to investigate the comprehensive efficacy of PF and SPF. The internal correlations among these results, together with the influence of salt-processing, were then comprehensively analysed to reveal the mechanism of salt-processing on PF.

## Results

### Quantitative analysis of bioactive components in PF and SPF extract

The contents of psoralen, neobavaisoflavone, corylifolin, corylin, psoralidin, isobavachalcone, bavachinin and corylifol A in PF and SPF extracts were shown in Table 1. The contents of all analytes obviously increased to some extent after salt-processing. Detailed methodological content can be found in the Supplementary Information.

**Table 1:** Quantitative analysis results for all analytes in PF and SPF extract.

No.	Analytes	PF (µg/g)	SPF (µg/g)	Percentage of increase after processing (%)
1	Psoralen	292.44 ± 2.43	392.08 ± 4.61	34.07
2	Neobavaisoflavone	263.91 ± 4.73	365.24 ± 6.26	38.40
3	Corylifolin	124.38 ± 2.35	181.61 ± 1.62	46.01
4	Corylin	26.52 ± 0.38	35.24 ± 0.38	32.89
5	Psoralidin	70.95 ± 1.25	94.82 ± 1.24	33.64
6	Isobavachalcone	236.71 ± 3.61	336.20 ± 6.26	42.03
7	Bavachinin	389.32 ± 3.59	504.22 ± 7.21	29.51
8	Corylifol A	201.61 ± 2.94	274.98 ± 2.76	36.39

### Pharmacokinetics study

#### Specificity

There was no endogenous interference in the retention time of psoralen, neobavaisoflavone, corylifolin, isobavachalcone, bavachinin, corylifol A and chloramphenicol (IS).

#### Linearity and lower limit of quantification (LLOQ)

The regression equations, linearity ranges, correlation coefficients and LLOQs for the eight analytes were summarized in Supplementary Table S3. The correlation coefficients of linearity were higher than 0.998, demonstrating that all calibration curves were linear over the entire calibration range. The LLOQs ranged from 0.136 to 0.204 ng/mL, suggesting that the method was sensitive enough for all of the analytes in plasma.

#### Precision, accuracy, extraction recovery, matrix effect and stability

The precision, accuracy, extraction recovery, matrix effect and stability of the eight analytes were summarized in Supplementary Tables S4, S5, and all results were within the prescribed range.

#### Pharmacokinetic analysis

The validated method was applied to the pharmacokinetic study of analytes after oral administration of PF and SPF extract. All the analytes were analysed with the two-compartment pharmacokinetic model. The mean plasma concentration-time curves (*n* = 6) were shown in Supplementary Fig. S1, and the corresponding pharmacokinetic parameters were listed in Table 2. The absorption of psoralen, isobavachalcone, bavachinin and corylifol A increased significantly after salt-processing, while neobavaisoflavone and corylifolin presented unclear variation trends. Except for psoralen, the SPF group exhibited a higher *t*_max_ and a lower *C*_max_ than the PF group.

**Table 2:** Pharmacokinetic parameters of analytes in rat plasma (*n* = 6, mean ± SD).

Gro	Parameter	Psoralen	Neobavaiso-flavone	Corylifolin	Isobavachal-cone	Bavachinin	Corylifol A
PF	*AUC*_(0–t)_ (ug/L*h)	172.36 ± 24.55	42.51 ± ± 6.57	75.91 ± 9.22	32.70 ± 7.40	30.17 ± 9.41	10.53 ± 1.36
	MRT _(0–t)_ (h)	8.64 ± 0.33	6.67 ± 1.77	10.80 ± 0.57	8.68 ± 2.27	8.928 ± 3.273	7.16 ± 0.93
	*t*_1/2_ (h)	5.39 ± 2.46	12.07 ± 12.43	125.96 ± 122.9	28.69 ± 36.33	11.25 ± 11.01	14.587 ± 12.72
	*t*_max_ (h)	5 ± 0	0.25 ± 0	0.25 ± 0	0.25 ± 0	0.25 ± 0	0.25 ± 0
	*C*_max_ (ug/L)	20.68 ± 5.79	38.16 ± 36.04	72.97 ± 10.84	23.9 ± 10.18	23.15 ± 16.08	8.59 ± 3.73
SPF	*AUC*_(0–t)_ (ug/L*h)	2860.3 ± 997.9	38.16 ± 36.04	72.97 ± 10.84	55.93 ± 52.36	51.98 ± 14.55	22.21 ± 21.60
	MRT _(0-t)_ (h)	7.84 ± 0.77	6.59 ± 2.96	10.22 ± 0.47	9.30 ± 3.06	6.62 ± 0.64	6.82 ± 1.17
	*t*_1/2_ (h)	5.17 ± 1.07	8.33 ± 3.73	68.99 ± 39.89	16.06 ± 14.81	6.20 ± 1.4	9.46 ± 7.5
	*t*_max_ (h)	5.8 ± 1.10	0.25 ± 0	1.25 ± 1.90	2.54 ± 3.68	1.08 ± 1.92	0.25 ± 0
	*C*_max_ (ug/L)	331.06 ± 181.07	18.62 ± 3.91	11.01 ± 2.15	22.24 ± 11.05	14.40 ± 2.35	12.61 ± 3.99

### Metabolomics study

#### Metabolic changes in response to PF and SPF

**Figure 1:**
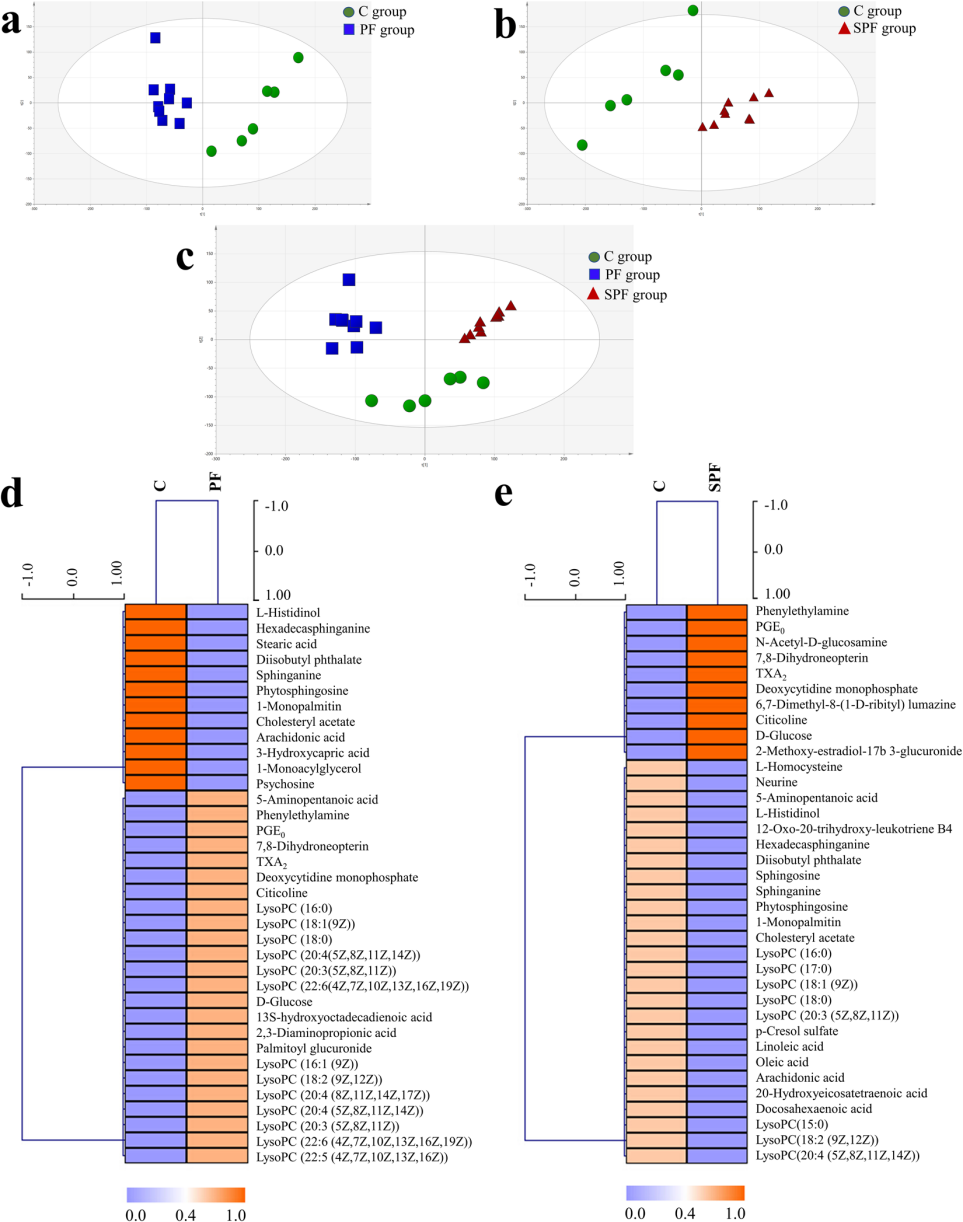
PCA score scatter plots and heatmaps obtained from the control group and treatment groups. PCA score scatter plots obtained from C and PF group (**a**), C and SPF group (**b**), C, PF and SPF group (**c**), heatmaps of disturbed metabolites responding to PF exposure (**d**) and SPF exposure (**e**).

The normalized data from ESI^−^ and ESI^+^ modes were merged and input into SIMCA-P software (version 13.0, Umetrics AB) for multivariate statistical analysis. First, principal component analysis (PCA) was performed to investigate the holistic metabolic variations in the PF and SPF groups. In the process of analysis, no observations or variables had missing values exceeding the missing value tolerance. The PCA score scatter plots exhibited a clear separation between the control (C) and PF groups (R^2^X = 0.475, Q^2^ = 0.243, Fig. 1a), as well as the C and SPF groups (R^2^X = 0.534, Q^2^ = 0.314, Fig. 1b). A Student’s *t*-test on PCA scores was also carried out, and the *P* value for the C *vs*. PF group was 0.04 and that for the C *vs*. SPF group was 0.01. Therefore, the differences observed in PCA score scatter plots (Fig. 1a,b) were significant, i.e., the plasma metabolic patterns of rats were significantly changed by PF and SPF, respectively. Furthermore, an obvious grouping tendency between the PF and SPF groups was displayed in Fig. 1c, which confirmed the different metabolic phenotypes between PF- and SPF-treated rats (R^2^X = 0.585, Q^2^ = 0.395).

#### Potential biomarkers for PF and SPF

The orthogonal partial least squares discriminant analysis (OPLS-DA) model was constructed to compare the differences in metabolism. As shown in Supplementary Fig. S2a,b, clear separations were observed between the C group and PF group (R^2^X = 0.51; R^2^Y = 0.99; Q^2^ = 0.953) as well as the C group and SPF group (R^2^X = 0.582; R^2^Y = 0.995; Q^2^ = 0.974). The *P* values provided by cross-validated ANOVA (*P*_CV-ANOVA_) were 1.29 × 10^−6^ and 1.34 × 10^−8^ for the above two OPLS-DA models, respectively, which conformed the significance of the models. For further validation, permutation tests with 200 iterations were performed. The goodness of fit of the randomly permuted models was compared with that of the original model. As shown in the validation plots for OPLS-DA models (Supplementary Fig. S2c,d), all permuted R^2^ and Q^2^ values to the left were lower than the original point to the right, and the blue regression line of Q^2^ points had a negative intercept. Therefore, the original two OPLS-DA models were all valid. The five-pointed star symbols in Supplementary Fig. S2e,f represent the significantly changed metabolites in the PF group and SPF group. The metabolites could be approved as potential biomarkers after being screened by adjusted *P* values (*P* < 0.05) and variable importance for the projection (VIP) values (VIP > 1.5). The structures of potential biomarkers were then identified on the basis of data information, including MS/MS fragment, accurate mass and the origin in the Human Metabolome Database (http://www.hmdb.ca/); Metlin (https://metlin.scripps.edu/); MassBank (http://www.massbank.jp/) and KEGG (http://www.kegg.jp/). Further confirmation was achieved by comparing retention times and MS/MS fragment patterns with reference standards when necessary. Based on the above strategies, a total of 33 and 36 biomarkers in response to PF and SPF exposure, respectively, were obtained and were listed in Supplementary Tables S6 and S7.

**Figure 2:**
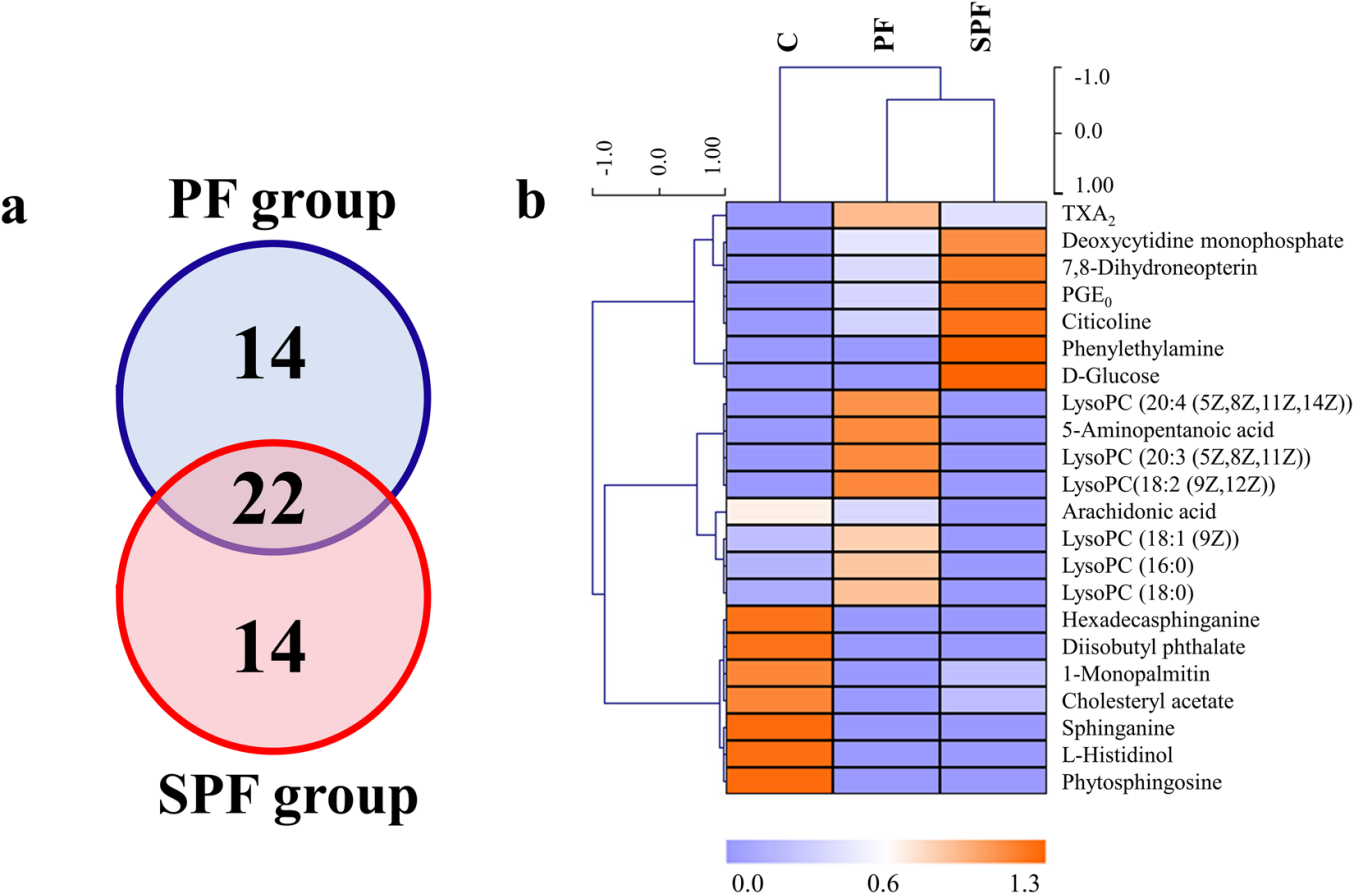
A panel of 22 common disrupted metabolites between the PF and SPF groups. (**a**) Venn diagram of metabolite number exhibiting the significant difference between PF and SPF group, (**b**) heatmap of 22 common disrupted metabolites. The colors from blue to orange indicate the increasing levels of metabolites.

A heatmap was applied to intuitively inspect the variation tendencies of metabolite levels between the C group and the treatment groups. Compared with the C group, 24 metabolites were upregulated and 12 metabolites were downregulated in the PF group (Fig. 1d); however, 10 were upregulated and 26 were downregulated in the SPF group (Fig. 1e). Among the 22 common disrupted metabolites, 5-aminopentanoic acid and lysophosphatidylcholines (lysoPCs) presented a converse trend that fell in response to SPF and rose in response to PF. Additional, the levels of thromboxane A2 (TXA_2_), deoxycytidine monophosphate, 7,8-dihydroneopterin, 13,14-dihydro-prostaglandin E1 (PGE_0_), citicoline, phenylethylamine and D-glucose were all increased under the two treatments, and the effect of SPF was stronger than that of PF. The other eight metabolites, including arachidonic acid, hexadecasphinganine, diisobutyl phthalate, 1-monopalmitin, cholesteryl acetate, L-histidinol, sphinganine and phytosphingosine displayed the same decreasing trend (Fig. 2).

#### Metabolic pathway analysis for PF and SPF

**Figure 3:**
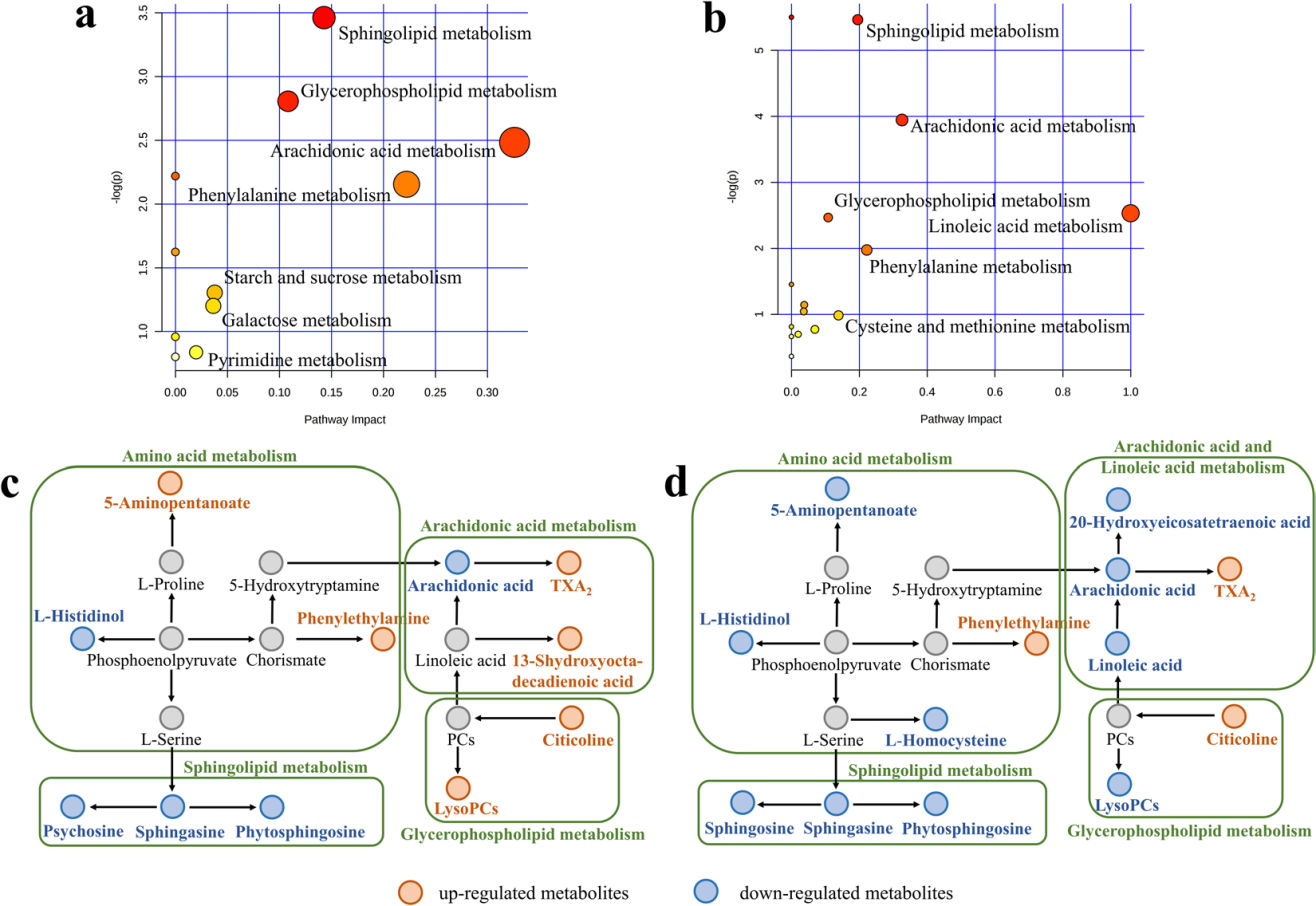
Metabolic pathway enrichment analysis and networks responding to PF and SPF exposure. Pathway enrichment analysis of PF (**a**) and SPF (**b**), metabolic pathway networks responding to PF exposure (**c**) and SPF exposure (**d**).

Further analysis of the significant pathways influenced by PF and SPF was conducted through application of the MetaboAnalyst online database. As shown in Fig. 3a,b, sphingolipid metabolism, glycerophospholipid metabolism, arachidonic acid metabolism and phenylalanine metabolism pathways were influenced by both PF and SPF. The starch and sucrose metabolism, galactose metabolism and pyrimidine metabolism pathways were influenced only by PF, while linoleic acid metabolism, cysteine and methionine metabolism pathways were influenced only by SPF. To investigate the latent relationships among the disrupted pathways, correlation network diagrams were constructed based on the above results and the KEGG pathway database. As shown in Fig. 3c,d, the effects of PF and SPF on most pathways were similar, except for glycerophospholipid metabolism, which was upregulated by PF and downregulated by SPF.

## Discussion

### Effects of salt-processing on chemical composition

The levels of bioactive components in SPF extract were 30–50% higher than those in PF extract. This could be attributed to the following two aspects. (1) PF expanded in volume, and the internal micro-structure became loose due to the heating effect during salt-processing. Hence, it was easier for water to permeate into medicinal materials, dissolve the bioactive components, and then diffuse smoothly into the decoction in the boiling process. (2) Under the effects of high temperature and sodium ions, linoleic acid, oleic acid, palmitic acid and stearic acid in PF could generate sodium linoleate, sodium oleate, sodium palmitate and sodium stearate, which were substances possessing surface activity^14^. Surfactants could form micelles in water, which increased the solubility of the other slightly soluble components in PF^15^. Therefore, the overall level of bioactive components in extract increased significantly after salt-processing.

### Effects of salt-processing on absorption characteristics

*AUC*, *C*_max_ and *t*_max_ are often employed to characterize the degree, intensity and speed of absorption, respectively. The pharmacokinetic results suggested that salt-processing was conducive to improving the absorptions of most bioactive components and alleviating the intensity and speed of action. This principle could be demonstrated by the following three points. (1) The surfactants generated during salt-processing could dissolve the membrane lipid of intestinal mucosal epithelial cells, thereby enhancing the permeability of the membrane and absorption of the components^16^. (2) The complexes formed by surfactants and certain components changed the physical and chemical properties of the original compounds, such as solubility, molecular size, diffusion velocity and oil/water partition coefficient, and enhanced the associated membrane permeability^17,18^. (3) PF has the function of accelerating gastrointestinal peristalsis. Administration of PF extract would cause more bioactive components to gather at the absorption site simultaneously and result in shorter residence time. Therefore, the corresponding results of higher *C*_max_, shorter *t*_max_ and lower *AUC* in the PF group could be explained. In contrast, SPF could inhibit gastrointestinal motility efficaciously, slowing down the movement of bioactive components in the gastrointestinal tract and resulting in longer *t*_max_, lower *C*_max_ and higher *AUC*^19^. Moreover, this also provided theoretical support for the enhanced anti-diarrhoeal effect of PF after salt-processing.

### Effects of salt-processing on comprehensive efficacy

#### Enhancing the efficacy

PF is usually used for improving the osteoporosis and diarrhoea in clinic. Before the present study, we investigated the effects of PF and SPF in both an osteoporosis model and a diarrhoea model. The results showed that SPF was more efficacious than PF in treating both osteoporosis and diarrhoea (detailed information is shown in Supplementary Information, section 2 and section 3). In the present study, the levels of deoxycytidine monophosphate, 7,8-dihydroneopterin, PGE_0_, citicoline, phenylethylamine and D-glucose in SPF group were higher than that in PF group (Fig. 2b). Therefore, SPF possessed a stronger effect than PF, which might be related to the increased contents of bioactive components in extract and the enhanced absorption *in vivo* after salt-processing.

#### Alleviating the toxicity

Prostaglandin I2 (PGI_2_) can inhibit platelet aggregation and dilate blood vessels. TXA_2_, a metabolite in arachidonic acid metabolism, possesses activity that is opposite to that of PGI_2_^20^. TXA_2_ promotes thrombus formation and causes serious injury to renal function^21^. Therefore, the PGI_2_/TXA_2_ value plays a key role in modulating renal blood flow and function, and is often used as an important indicator of renal injury^22,23^. Due to the similar effect with PGI_2_, the PGE_0_/TXA_2_ value can be a substitute for the PGI_2_/TXA_2_ value. As shown in Fig. 2b, the PGE_0_/TXA_2_ value in the SPF group was higher than that in the PF group, suggesting that the toxic side effect of PF on the renal system could be alleviated by salt-processing.

LysoPCs are generated from phosphatidylcholines by the actions of lecithin-cholesterol acyltransferase (LCAT). High levels of lysoPCs can induce endothelial dysfunction and atherosclerosis by regulating vascular tension^24,25^ and induce cytotoxicity by destroying cell membrane phospholipids. LysoPCs can also induce or aggravate renal cell damage by accelerating the exchange rate between cytomembrane and cytoplasm^26^. Compared with the C group, the levels of lysoPCs were upregulated in the PF group and showed a downward trend in the SPF group (Fig. 2b). These results indicated that the toxic side effects of PF on the cardiovascular and renal systems could be alleviated by salt-processing. All alleviation in toxicity might be attributed to the loss of volatile components during the heating process of salt-processing. Bakuchiol, as an example, is one of the major volatile components in PF^27^ and achieves the maximum decrease in relative content after salt-processing^28^. It has been reported that bakuchiol exhibited cytotoxicity and its metabolites were against human kidney-2 cell line^29^. Accordingly, the lower the amounts of volatile components were, the weaker the toxic side effects of PF would be.

#### Maintaining the efficacy

Sphinganine and phytosphingosine are sphingolipids, the free types of which cause growth inhibition and cytotoxicity in renal cells. Under the actions of different enzymes, sphinganine and phytosphingosine are converted into other important metabolites in sphingolipid metabolism, such as ceramide and sphingosine-1-phosphate^30,31^. Ceramide, an important second messenger, plays a key role in apoptosis^32^. Studies have shown that long chain ceramide can induce osteoblast apoptosis^33^. As shown in Fig. 2b, both PF and SPF inhibited the levels of sphinganine, phytosphingosine and the associated sphingolipid metabolism. I.e., PF and SPF could reduce the level of ceramide *in vivo*, inhibit osteoblast apoptosis and prevent osteoporosis, by inhibiting sphingolipid metabolism. Additionally, PF and SPF exhibited similar impacts on arachidonic acid, hexadecasphinganine, diisobutyl phthalate, 1-monopalmitin, cholesteryl acetate and L-histidinol. These results lead to the hypothesis that the type and/or contents of certain components might be altered in the salt-processing procedure, but the basic chemical composition was preserved. Correspondingly, PF and SPF exert similar effects in some respects.

### The comprehensive mechanism of salt-processing on PF

**Figure 4:**
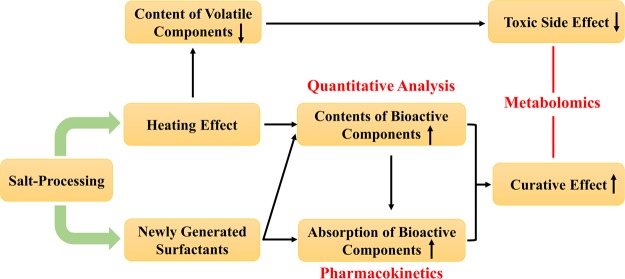
Comprehensive mechanism of salt-processing on PF.

The two main steps of salt-processing were (i) infiltration by salt solution and (ii) stir-frying over a flame. The heat from the flame made the internal structure of PF loose and caused the volatile components to vaporize. The combined effects of high temperature and sodium ions initiated the emergence of surfactants, which could form micelles in water, combine with some components to generate complexes and dissolve the membrane lipid of intestinal mucosal epithelial cells. The loosened structure and solubilization effect from micelles were crucial to the increased content of bioactive components in extract. The newly generated complexes and membrane lipid solubility of surfactants made it easy for the bioactive components to pass through the absorption barrier. Therefore, both the enhancements in the content and in the absorption of the bioactive components contributed to the strengthened efficacy of SPF. Moreover, volatilization loss that occurred during the heating operation was the principal reason for the alleviated toxic effects of SPF. The comprehensive mechanism of salt-processing on PF was simplified in Fig. 4.

## Conclusion

Research based on quantitative analysis, pharmacokinetics and metabolomics was successfully performed to explore the comprehensive effects of salt-processing on PF. The internal correlations among chemical composition, absorption characteristics and action mechanism, together with the influence of salt-processing, have led to the discovery of the salt-processing mechanism. In summary, the effects of heating and newly generated surfactants in the salt-processing procedure are the primary cause of the changes in chemical composition and absorption characteristics, as well as the subsequent enhanced efficacy and minor toxicity of SPF. Furthermore, this integrated novel strategy is a feasible approach for future mechanism study of herb-processing.

## Materials and Methods

### Chemicals and reagents

Standards of psoralen, corylifolin, corylin, bavachinin and chloramphenicol (IS) were purchased from Shanghai Yuanye Bio-Technology Co., Ltd. (Shanghai, China). Standards of neobavaisoflavone, psoralidin, isobavachalcone and corylifol A were purchased from Chengdu Herbpurify Co., Ltd. (Sichuan, China). The purity of all the standards were above 98%, with the chemical structures displayed in Supplementary Fig. S3.

HPLC grade acetonitrile was purchased from Fisher Chemical (USA). LC-MS grade formic acid was purchased from Anaqua Chemicals Supply Inc. (USA). Deionized water was prepared with Molecular Water Purification system. PF were purchased from Bozhou herbal medicine market (Anhui, China) and authenticated by Professor Cheng-Ming Dong and Sui-Qing Chen (Henan University of Chinese Medicine, Zhengzhou, China). Voucher specimens were deposited in Henan University of Chinese Medicine. SPF were processed according to our previous study^34^. Briefly, the PF were mixed with 20% (20:100, salt-water, w/v) salt solution (10:1, PF-salt solution, w/v) and placed in a closed container until salt solution was absorbed completely by PF. Then the moistened PF were stir-fried in a metallic pan over a low flame at 130 ± 20 °C for 8.5 min. After cooling, the SPF were dried in vacuum drying oven at 40 °C for 24 h.

### Extraction

A total of 500 g PF was decocted with boiling water twice (1:8 and 1:6), for 1 h each time. The same operation was carried out with 500 g SPF. Then, the combined decoction was concentrated and dried in vacuum to obtain PF extracts (48.1 g) and SPF extracts (62.6 g), respectively. The extracts were stored at 4 °C before analysis and administration.

### Animals

Sprague-Dawley rats (weighing 240~260 g, male) were obtained from the Shandong Laboratory Animal Centre (Shandong, China, Certificate No. SYXK 2015-0005). All animals were housed at 20 ± 2 °C and humidity of 60 ± 10% with a 12 h light/12 h dark cycle and free access to water and food. All animal experiments were approved by the Experimental Animal Ethics Committee of Henan University of Chinese Medicine. In addition, all methods were performed in accordance with the relevant guidelines and regulations.

### Quantitative analysis of bioactive components in PF and SPF extracts

20 mg PF and SPF extract were dissolved in 50% methanol solution (50:50, methanol-water, v/v), respectively. The solutions were ultrasonically processed (for 10 min), moderately diluted and centrifuged at 20,000 × g for 10 min. Then, 2 μL of the supernatants were injected into UPLC-Q-TOF/MS for analysis. Detailed methodological content can be found in the Supplementary Information.

### Pharmacokinetics study

#### LC system and mass spectrometry

Separation was performed by the Dionex UltiMate 3000 UPLC system (Thermo Scientific, USA) and screened with ESI-Q-TOF/MS. The LC analysis was performed on an Acclaim^TM^ RSLC 120 C_18_ column (2.2 µm, 2.1 × 100 mm; Thermo Scientific, USA) at 40 °C. The mobile phase was composed of solvent A (0.1% formic acid-water) and solvent B (acetonitrile) with a gradient elution (0–2 min, 95–30% A; 2–5 min, 30–15% A; 5–8 min, 15–2% A). The sample manager temperature was set at 4 °C and the flow rate was 0.3 mL/min.

The MS analysis was performed on a maXis HD Q-TOF/MS (Bruker, Germany) using an ESI source. The capillary voltage was 3200 V and 3500 V in negative and positive mode, respectively. The scanning mass range (*m/z*) was 50–1500 and spectra rate was 1.00 Hz. The nebulizer pressure, dry gas temperature and continuous dry gas flow rate were set at 2.0 Bar, 230 °C, and 8 L/min, respectively.

#### Calibration solutions and quality control sample

The calibration solutions of psoralen, neobavaisoflavone, corylifolin, corylin, psoralidin, isobavachalcone, bavachinin and corylifol A were prepared by adding 20 µL mixed standard solutions (refer to Supplementary Information) and 10 µL IS to blank plasma. Quality control (QC) samples were prepared at the concentrations of 1.77, 17.7 and 177 ng/mL for psoralen, 1.65, 16.5 and 165 ng/mL for neobavaisoflavone, 1.82, 18.2 and 182 ng/mL for corylifolin, 1.46, 14.6 and 146 ng/mL for corylin, 2.04, 20.4 and 204 ng/mL for psoralidin, 1.37, 13.7 and 137 ng/mL for isobavachalcone, 1.36, 13.6 and 136 ng/mL for bavachinin, 1.62, 16.2 and 162 ng/mL for corylifol A. All solutions were stored at 4 °C before analysis.

#### Sample preparation

Prior to analysis, plasma samples were thawed in ice-water. Each plasma sample (100 µL) was mixed with 400 µL cold acetonitrile and 10 µL IS in a centrifuge tube. The mixture was vortexed for 3 min and centrifuged at 12,000 × g for 10 min to precipitate protein. Then, the supernatant was transferred to another clean tube and evaporated to dryness under N_2_ at 40 °C. Each residue was reconstituted with 200 µL acetonitrile for analysis.

#### Method validation

(1) Specificity: The specificity was evaluated by comparing the blank plasma samples with spiked bio-samples of psoralen, neobavaisoflavone, corylifolin, isobavachalcone, bavachinin, corylifol A and IS, and the actual plasma samples after administration of PF and SPF extract. **(**2) Linearity and sensitivity: Calibration curves were prepared by plotting the measured peak area ratios of standards/IS versus concentrations. Then, linear regressions were carried out and correlation coefficients were obtained. The LLOQ for analytes were the lowest concentrations with a signal-to-noise ratio ≥10, while the precision and accuracy were within ±20%. (3) Precision and accuracy: The QC samples were analysed in six replicates on the same day for three consecutive days to evaluate the precision and accuracy. Relative standard deviation (RSD) and accuracy value were utilized to evaluate the precision and accuracy, respectively. The receivable values for validation of precision and accuracy were ≤15% and 85–115%, respectively. (4) Recovery and matrix effect: The extraction recoveries of analytes were assessed by comparing the peak areas of the spiked plasma samples (blank plasma spiked with low, middle, and high levels standards) with the ordinary prepared QC samples. The matrix effect was evaluated by comparing the peak areas of the post-extraction blank plasma samples spiked with standards with those obtained from mobile phase spiked with standards at low, middle, and high levels. (5) Stability: The stability of analytes in plasma samples was investigated at low, middle, and high QC levels under different conditions: three cycles of freeze-thaw, storage at −80 °C for 15 days and room temperature for 6 h. The receivable RSD values for stability were within ±15%.

#### Pharmacokinetic analysis

The rats were randomly divided into two groups (*n* = 6). PF and SPF groups were orally administered with PF extracts (4 g/kg) and SPF extracts (4 g/kg), respectively. 500 µL blood samples were collected into heparinized tubes from the ophthalmic venous plexus at 0, 0.08, 0.17, 0.25, 0.5, 0.75, 1, 1.5, 2, 3, 5, 7, 9 and 24 h after administration. The samples were promptly centrifuged at 12,000 × g for 5 min and the supernatants were stored at −20 °C before analysis. The pharmacokinetic parameters, including *AUC*, MRT, *C*_max_, *t*_max_, and *t*_1/2_ were calculated by DAS software (version 2.0, China State Drug Administration).

### Metabolomics study

#### Administration

The rats were randomly divided into 3 groups (*n* = 10): control group (C), PF group and SPF group. The C group was administered with water. The PF and SPF groups were orally administered with PF extract (2 g/kg) or SPF extract (2 g/kg) once per day for four weeks, respectively. All animals were sacrificed after collection of blood from the abdominal aorta. The blood samples were promptly centrifuged at 3,000 × g for 10 min and the supernatants were stored at −20 °C before analysis.

#### Statistical analysis

The raw data obtained from UPLC-Q-TOF/MS were background noise subtracted, peak aligned and calibrated by Data Analysis (version 4.1, Bruker). The generated data list was opened in Profile Analysis (version 2.1, Bruker) for bucketing, normalization and bucket filtering. The rectangle bucketing was performed using the following settings: retention time range 18 ~ 480 s, mass range (*m/z*) 60 ~ 700 Da, retention time width 20 s, and mass width (*m/z*) 1 Da. The “Sum of bucket values in analysis” option was used for normalization. The value count of group attribute within bucket ≥20% was set for the bucket filter. The consequent “bucket table” was then imported to SIMCA-P software (version 13.0, Umetrics AB) for multivariate statistical analysis. PCA was first performed as the unsupervised method for outlier identification^35^. Subsequently, the OPLS-DA was implemented as the supervised method to identify potential biomarkers. The data were pre-treated using unit variance scaling and mean-centring before PCA, and Pareto scaling before OPLS-DA. The missing value tolerance was set at variable 50% and observation 50%. In SIMCA-P software, the OPLS-DA models were validated using 7-fold cross-validation and permutation testing. The *P* value provided by cross-validated ANOVA (*P*_CV-ANPVA_) was used to estimate the model significance^36^. Model variance and predictability were assessed by R^2^ and Q^2^ values. The R^2^X and R^2^Y values represented the explained variation in X and Y matrices, respectively^37^. Customarily, permutation testing with 200 iterations was also performed for further validation^38^. In addition to the multivariate statistical analysis, the Student’s *t*-test was also applied to measure the significance of the differences observed in PCA score scatter plots, as well as the significance of each metabolite. The *P* values across all metabolites within each comparison were adjusted by a false discovery rate method to account for multiple testing. The adjusted *P* values (*P* < 0.05) and VIP values (VIP > 1.5) were utilized together for filtering biomarkers^39^.

Moreover, hierarchical cluster analysis and heatmaps were performed by MeV software (version 4.8.0). Correlation networks for disturbed metabolic pathways were constructed on the base of the MBRole database^40^, KEGG and MetaboAnalyst.

## Supplementary information

Supplementary Information

## Data Availability

The datasets generated during and/or analysed during the current study are available from the corresponding author on reasonable request.
