# Supplementary material for: Quantitative analysis, pharmacokinetics and metabolomics study for the comprehensive characterization of the salt-processing mechanism of Psoraleae Fructus
Source: Sci Rep. 2019 Jan 24;9:661. doi: 10.1038/s41598-018-36908-w (PMC6345873; doi:10.1038/s41598-018-36908-w)
Supplement: Supplementary file 1 — Supplementary Information [file 41598_2018_36908_MOESM1_ESM.pdf]

Quantitative analysis, pharmacokinetics and metabolomics study for the comprehensive characterization of the salt-processing mechanism of Psoraleae Fructus

Kai Li[*] [1,2], Ning Zhou[1], Xiao-Ke Zheng[1], Wei-Sheng Feng[*] [1,2], Fei Li[3], Zhen-Ling Zhang[1], Ya-Qi Lu[1]

[1] College of Pharmacy, Henan University of Chinese Medicine, Zhengzhou 450046, China

[2] Collaborative Innovation Center for Respiratory Disease Diagnosis and Treatment & Chinese Medicine Development of Henan Province, Zhengzhou 450046, China

[3] State Key Laboratory of Natural Medicines, China Pharmaceutical University, Nanjing 210009, China

E-mail addresses: cpulikai@163.com (K. Li), zhoun0813@163.com (N. Zhou), zhengxk.2006@163.com (X.-K. Zheng), fwsh@hactcm.edu.cn (W.-S. Feng), lifeicpu@163.com (F. Li), zhangzl6758@163.com (Z.-L. Zhang), 1538561584@qq.com (Y.-Q. Lu).

Kai Li, Email: cpulikai@163.com, Tel: 86-371-65962746, Fax: 86-371-65945879.

[*] Corresponding authors.

1. Quantitative analysis of bioactive components in PF and SPF extracts

Results

Linearity and sensitivity. The regression equations, linearity ranges, correlation coefficients and the lower limit of quantifications (LLOQs) for the eight analytes were presented in Supplementary Table S1. The correlation coefficients of linearity were higher than 0.998, demonstrating that all calibration curves were linear over the entire calibration range. The LLOQs ranged from 1.36 to 2.04 ng/mL, suggesting that the method was sensitive enough for the analytes in PF and SPF extracts.

Precision, repeatability, stability and recovery. The intra- and inter-day precision, repeatability, stability and recovery of the eight analytes were summarized in Supplementary Table S2. The relative standard deviation (RSD) values of intra- and inter-day precision ranged from 0.93% to 1.80%, and from 1.09% to 2.27%, respectively. The repeatability and stability RSD values were 1.12-1.96% and 0.84-1.75% respectively. The recoveries of all analytes varied from 99.64% to 99.98% with RSD values from 1.07% to 1.66%.

Methods

LC system and mass spectrometry. Separation was performed by the Dionex UltiMate 3000 UPLC system (Thermo Scientific, USA) and screened with ESI-Q-TOF/MS. The LC analysis was performed on an Acclaim^{TM} RSLC 120 C_{18} column (2.2 µm, 2.1×100 mm; Thermo Scientific, USA) at 40 °C. The mobile phase was composed of solvent A (0.1% formic acid-water) and solvent B (acetonitrile) with a gradient elution (0-3 min, 90-55% A; 3-13 min, 55-20% A; 13-17 min, 20-5% A). The sample manager temperature was set at 4 °C and the flow rate was 0.3 mL/min.

MS analysis was performed on a maXis HD Q-TOF/MS (Bruker, Germany) using an ESI source. The capillary voltage was 3200 V and 3500 V in negative and positive mode, respectively. The scanning mass range (*m/z*) was 50-1500 and spectra rate was 1.00 Hz. The pressure of the nebulizer, dry gas temperature and continuous dry gas flow rate was set at 2.0 Bar, 230 °C, and 8 L/min,

respectively.

Calibration solutions and sample preparation. Stock solutions (1 mg/mL) of psoralen, neobavaisoflavone, corylifolin, corylin, psoralidin, isobavachalcone, bavachinin and corylifol A were prepared in methanol, respectively. They were first mixed in a high concentration. Then, the mixed solutions were serially diluted with methanol to achieve the final linearity concentrations of 1.77-885 ng/mL for psoralen, 1.65-412.5 ng/mL for neobavaisoflavone, 1.82-910 ng/mL for corylifolin, 1.46-365 ng/mL for corylin, 2.04-510 ng/mL for psoralidin, 1.37-342.5 ng/mL for isobavachalcone, 1.36-340 ng/mL for bavachinin and 1.62-405 ng/mL for corylifol A. All solutions were stored at 4 °C before analysis.

20 mg PF and SPF extract were dissolved in 50 % methanol solution (50:50, methanol-water, v/v), respectively. The solutions were ultrasonically processed (for 10 min), moderately diluted and centrifuged at 20, 000 g for 10 min. Then, 2 μL of the supernatants were injected into UPLC-Q-TOF/MS for analysis, respectively.

Method validation. *Linearity and sensitivity.* Calibration curves were prepared by plotting the measured peak areas versus concentrations of calibration standards. Then, linear regressions were carried out and correlation coefficients (r) were obtained. The LLOQs for analytes were the lowest concentrations with a signal-to-noise ratio ≥ 10, while the precision and accuracy were within ±20%.

Precision, repeatability and stability. The mixed standard solution at a middle level concentration was analysed in six replicates within one day for three consecutive days to evaluate the intra-day and inter-day precision, respectively. The repeatability of this method was evaluated by analysis of six replicates of the same batch extracts. For stability testing, the sample solution was examined at room temperature for 12 consecutive hours and injections were performed at 0, 2, 4, 6, 8 and 10 h. RSD was utilized to evaluate the precision, repeatability and stability of this method.

Recovery. The samples were spiked with 80%, 100%, and 120% of known amounts of standards respectively, achieving three levels of concentration (low, middle, and high). The spiked samples of

each level were analysed in triplicate. The ultimate result was evaluated by recovery (recovery (%) = (detected amount - original amount) / spiked amount × 100%) and RSD.

2. Effects of PF and SPF on the osteoporosis model

Results

Biochemical analysis and histopathological observations. Alkaline phosphatase (ALP) is a reliable index for osteoblast activity. The wet and/or dry bone coefficient of the left femur can reflect the severity of bone loss. Compared with the C group, the level of serum ALP, the wet bone coefficient of the left femur and the dry bone coefficient of the left femur decreased significantly in the OST group, indicating the excellence of the model. As shown in Supplementary Table S8, ED (positive drug), PF and SPF could all improve the bone status to some extent. SPF exhibited obviously stronger efficacy than PF in treating osteoporosis.

A similar phenomenon also appeared in the result of histopathological examination, as shown in Supplementary Fig. S4. Compared with the C group, the number of bone trabeculae was significantly reduced, the gap between trabeculae was larger and the bone trabeculae were thinner in the OST model group. ED, PF and SPF could all promote bone trabecular repair to some extent, and SPF performed better than PF.

Methods

Chemicals and reagents. Retinoic acid was obtained from Aladdin Reagent Co., Ltd. (Shanghai, China). Etidronate Disodium Tablets (ED) was obtained from CHIATAI TIANQING Pharmaceutical Group Co., Ltd. (Nanjing, China).

Animal handling. Fifty Sprague-Dawley rats (weighing 240 ~ 260 g, male) were randomly divided into 5 groups: control group (C), OST model group (OST), ED-treated group (ED, 40 mg/kg), PF extract-treated group (PF, 2 g/kg), SPF extract-treated group (SPF, 2 g/kg). All groups except the control group were intragastrically administered with retinoic acid (70 mg/kg/d) every morning and

the drug treatment every afternoon. Retinoic acid was administered for two weeks,[A] and the treating drugs were administered for four weeks. Meanwhile, the C group was orally administered with the same volume of distilled water. All rats were sacrificed after collection of blood from the abdominal aorta.

Biochemical and histological assessment. The alkaline phosphatase (ALP) serum level was measured using an ELISA kit in accordance with instructions (R&D Systems, Inc., USA). Bone loss was estimated by the wet bone coefficient of the left femur (left femur weight/body weight, g/100 g) and the dry bone coefficient of the left femur (dried left femur weight/body weight, g/100 g). Fresh right femur was put into 5% nitric acid solution for tissue slices preparation. Haematoxylin-eosin (HE) stained sections of the right femur were observed under microscope (ECLIPSE TS100, Nikon, Japan).

3. Effects of PF and SPF on the diarrhoea model

Results

Biochemical analysis and histopathological observations. Gastrin, a gastrointestinal hormone secreted by G cells in gastroduodenal mucosa, promotes gastric acid secretion and gastric antrum contraction. Motilin is a gastrointestinal hormone secreted by Mo cells which strongly stimulates gastric contraction and segmentation contraction. Gastrin and motilin are both important indicators in clinical diagnosis of diarrhoea. Compared with the C group, the levels of gastrin and motilin both increased significantly in the DIA group, indicating the excellence of the model. As shown in Supplementary Table S9, CD (positive drug), PF and SPF could all regulate the gastrointestinal hormone level to some extent. SPF exhibited obviously stronger efficacy than PF in treating diarrhoea.

Methods

Chemicals and reagents. Rheum officinale was obtained from Bozhou herbal medicine market (Anhui, China). Compound Diphenoxylate Tablets (CD) was obtained from Changzhou Kang Pu Pharmaceutical Co., Ltd. (Jiangsu, China).

Animal handling. Fifty Sprague-Dawley rats (weighing 240 ~ 260 g, male) were randomly divided into 5 groups: control group (C), DIA model group (DIA), CD-treated group (CD, 40 mg/kg), PF extract-treated group (PF, 2 g/kg), SPF extract-treated group (SPF, 2 g/kg). All groups except the control group were intragastrically administered with Rheum officinale extract (5 ml/kg/d) every morning and the drug treatment every afternoon. Rheum officinale extract was administered for two weeks, and the treating drugs were administered for three weeks. Meanwhile, the C group was orally administered with the same volume of distilled water. All rats were sacrificed after collection of blood from the abdominal aorta.

Biochemical and histological assessment. Serum levels of gastrin and motilin were measured using ELISA kits in accordance with instructions (R&D Systems, Inc., USA).

Supplementary Table S1

Regression equations, linearity ranges, correlation coefficients and LLOQs of all analytes.

No.	Analytes	Regression equation	Linear range (ng/mL)	*r*	LLOQ (ng/mL)
1	Psoralen	y = 6026.7x + 38934	1.77-885	0.9998	1.77
2	Neobavaisoflavone	y = 10209x - 3853.8	1.65-412.5	0.9997	1.65
3	Corylifolin	y = 7866x + 41246	1.82-910	0.9985	1.82
4	Corylin	y = 18256x + 25012	1.46-365	0.9998	1.46
5	Psoralidin	y = 6137.9x + 17347	2.04-510	0.9996	2.04
6	Isobavachalcone	y = 8418.2x - 15536	1.37-342.5	0.9996	1.37
7	Bavachinin	y = 10753x - 7167.2	1.36-340	0.9997	1.36
8	Corylifol A	y = 18288x + 39837	1.62-405	0.9997	1.62

Supplementary Table S2

Precision, repeatability, stability and recovery of all analytes.

No.	Analytes	Precision (RSD, %)		Repeatability (RSD, %)	Stability (RSD, %)	Recovery (%)	RSD (%)
		Intra-day	Inter-day				
1	Psoralen	0.97	1.97	1.17	0.84	99.64	1.66
2	Neobavaisoflavone	1.46	1.87	1.65	1.56	99.93	1.36
3	Corylifolin	1.00	1.23	1.96	1.16	99.87	1.39
4	Corylin	1.09	1.09	1.81	1.55	99.78	1.18
5	Psoralidin	1.80	2.27	1.78	1.75	99.87	1.14
6	Isobavachalcone	0.93	1.42	1.43	0.99	99.70	1.28
7	Bavachinin	1.25	2.26	1.29	1.06	99.98	1.07
8	Corylifol A	1.02	1.81	1.12	1.15	99.82	1.10

Supplementary Table S3

Regression equations, linearity ranges, correlation coefficients and LLOQs for all analytes in plasma.

No.	Analytes	Regression equation	Linear range (ng/mL)	*r*	LLOQ (ng/mL)
1	Psoralen	y = 0.2971x - 0.1596	0.177-177	0.9995	0.177
2	Neobavaisoflavone	y = 0.3217x + 0.0139	0.165-165	0.9984	0.165
3	Corylifolin	y = 0.1774x - 0.1732	0.182-182	0.9998	0.182
4	Corylin	y = 0.2419x + 0.1708	0.146-146	0.9998	0.146
5	Psoralidin	y = 0.0696x + 0.1017	0.204-204	0.9995	0.204
6	Isobavachalcone	y = 0.1847x - 0.0177	0.137-137	0.9984	0.137
7	Bavachinin	y = 0.1697x + 0.1515	0.136-136	0.9996	0.136
8	Corylifol A	y = 0.2227x + 0.3958	0.162-162	0.9997	0.162

Supplementary Table S4

Precision, accuracy, extraction recovery and matrix effect for all analytes in plasma.

Analytes	Concentration (ng/mL)	Intra-day		Inter-day		Extraction recovery		Matrix effect	
		Precision (RSD, %)	Accuracy (%)	Precision (RSD, %)	Accuracy (%)	Accuracy (%)	RSD (%)	Accuracy (%)	RSD (%)
Psoralen	1.77	7.11	95.67	10.74	94.50	90.98	7.8	94.08	6.1
	17.7	6.44	98.21	6.03	96.30	89.42	7.3	90.71	5.4
	177	6.30	97.45	5.99	97.48	90.15	8.3	89.46	7.5
Neobavaisoflavone	1.65	9.62	96.21	10.66	98.68	88.28	7.1	89.31	8.4
	16.5	9.92	95.17	5.73	94.37	91.61	9.6	92.26	8.7
	165	7.88	92.70	4.25	100.98	91.99	6.6	91.91	5.1
Corylifolin	1.82	9.41	91.21	6.81	102.53	87.18	7.2	88.13	4.3
	18.2	9.13	91.33	9.78	93.45	93.30	9.3	94.16	7.8
	182	6.51	95.95	7.04	97.24	90.71	5.8	91.34	6.2
Corylin	1.46	8.41	100.02	7.72	97.97	93.69	5.8	93.75	5.8
	14.6	8.50	105.07	8.10	97.83	88.63	5.3	89.88	4.4
	146	7.21	99.64	7.78	96.80	88.17	5.8	88.52	6.3
Psoralidin	2.04	7.39	98.18	8.24	101.86	92.21	7.2	95.31	6.9
	20.4	7.21	97.55	6.29	98.42	86.77	4.5	88.12	5.6
	204	5.49	94.07	5.33	99.18	87.12	7.0	87.68	5.3
Isobavachalcone	1.37	9.32	99.39	9.44	98.09	87.14	9.6	87.05	7.1
	13.7	6.77	104.27	5.25	96.60	91.92	5.7	93.40	6.3
	137	6.96	98.65	6.45	96.80	85.74	4.7	87.80	4.8
Bavachinin	1.36	9.54	97.00	7.82	101.54	92.71	7.5	93.28	6.5
	13.6	7.90	99.51	7.46	99.61	90.69	6.7	92.17	8.4
	136	6.34	95.31	5.80	100.24	90.28	5.8	90.64	6.1
Corylifol A	1.62	9.72	95.06	9.02	97.94	91.28	7.9	92.34	5.9
	16.2	8.05	95.13	8.61	93.02	87.90	7.0	88.79	6.1
	162	7.61	102.48	7.83	93.60	89.50	6.1	89.06	6.3

Supplementary Table S5

Stability for all analytes in plasma.

Analytes	Concentration (ng/mL)	Three freeze-thaw cycles		- 80 °C (15 d)		Room temperature (6 h)	
		Accuracy (%)	RSD (%)	Accuracy (%)	RSD (%)	Accuracy (%)	RSD (%)
Psoralen	1.77	101.08	6.23	100.29	6.27	99.00	7.63
	17.7	101.68	6.26	99.82	5.45	100.96	5.89
	177	99.56	4.47	99.72	6.72	94.87	5.32
Neobavaisoflavone	1.65	97.18	8.15	101.21	7.59	98.50	7.31
	16.5	98.06	7.64	99.52	6.70	98.16	6.41
	165	97.66	7.40	97.85	5.58	100.00	6.38
Corylifolin	1.82	98.43	7.40	97.80	7.19	97.02	8.58
	18.2	99.38	5.77	98.40	6.98	97.08	5.66
	182	100.80	6.42	99.65	5.87	98.15	5.94
Corylin	1.46	102.56	5.58	99.47	7.36	103.75	6.43
	14.6	101.67	5.67	98.59	5.62	96.80	5.31
	146	99.65	6.27	99.31	5.71	97.60	5.78
Psoralidin	2.04	96.23	8.65	96.93	8.36	97.59	6.77
	20.4	100.13	8.51	96.09	6.50	97.23	7.64
	204	98.21	6.38	99.28	6.06	95.62	4.54
Isobavachalcone	1.37	98.46	8.24	100.07	8.66	98.27	7.72
	13.7	99.49	5.62	99.50	6.85	99.79	6.65
	137	98.17	7.83	98.55	6.76	102.39	5.67
Bavachinin	1.36	99.47	6.05	96.52	8.66	97.93	8.79
	13.6	102.19	7.70	97.31	5.96	99.97	7.33
	136	99.26	6.56	97.40	6.27	98.51	5.85
Corylifol A	1.62	99.60	9.44	96.34	6.89	100.31	7.71
	16.2	100.84	7.88	97.70	6.02	97.42	6.43
	162	97.16	5.89	100.56	5.81	101.83	5.75

Supplementary Table S6

Potential biomarkers in response to PF exposure.

Mode	No.	Compound	Formula	t_R (min)	Determined *m/z*	Trend
ESI$^+$	1	5-Aminopentanoic acid	$C_5H_{11}NO_2$	0.8	118.0863	↑
	2	Phenylethylamine	$C_8H_{11}N$	14.8	122.0964	↑
	3	L-Histidinol	$C_6H_{11}N_3O$	12.2	124.0875	↓
	4	13,14-Dihydro-prostaglandin E1 (PGE_0)	$C_{20}H_{36}O_5$	13.8	190.1264	↑
	5	7, 8-Dihydroneopterin	$C_9H_{13}N_5O_4$	13.8	238.0940	↑
	6	Hexadecasphinganine	$C_{16}H_{35}NO_2$	10.5	256.2635	↓
	7	Stearic acid	$C_{18}H_{36}O_2$	10.5	267.2688	↓
	8	Diisobutyl phthalate	$C_{16}H_{22}O_4$	8.5	279.1591	↓
	9	Sphinganine	$C_{18}H_{39}NO_2$	6.8	302.3054	↓
	10	Phytosphingosine	$C_{18}H_{39}NO_3$	6.2	318.3003	↓
	11	1-Monopalmitin	$C_{19}H_{38}O_4$	10.8	331.2843	↓
	12	Thromboxane A2 (TXA_2)	$C_{20}H_{40}O$	13.8	338.3417	↑
	13	Deoxycytidine monophosphate	$C_9H_{14}N_3O_7P$	10.8	371.0727	↑
	14	Cholesteryl acetate	$C_{29}H_{48}O_2$	13.8	429.3727	↓
	15	Citicoline	$C_{14}H_{26}N_4O_{11}P_2$	13.8	471.1046	↑
	16	LysoPC (16:0)	$C_{24}H_{50}NO_7P$	8.2	496.3398	↑
	17	LysoPC (18:1 (9Z))	$C_{26}H_{52}NO_7P$	8.5	522.3554	↑
	18	LysoPC (18:0)	$C_{26}H_{54}NO_7P$	9.8	524.3711	↑
	19	LysoPC (20:3 (5Z,8Z,11Z))	$C_{28}H_{52}NO_7P$	7.8	546.3554	↑
	20	LysoPC (22:6 (4Z,7Z,10Z,13Z,16Z,19Z))	$C_{30}H_{50}NO_7P$	7.5	568.3398	↑
ESI$^-$	1	D-Glucose	$C_6H_{12}O_6$	1.1	225.0616	↑
	2	13S-hydroxyoctadecadienoic acid	$C_{18}H_{32}O_3$	7.8	295.2279	↑
	3	Arachidonic acid	$C_{20}H_{32}O_2$	10.5	303.2330	↓
	4	2,3-Diaminopropionic acid	$C_3H_8N_2O_2$	7.5	311.1685	↑
	5	3-Hydroxycapric acid	$C_{10}H_{20}O_3$	10.8	375.2752	↓
	6	1-Monoacylglycerol	$C_{21}H_{42}O_4$	12.1	403.3065	↓
	7	Psychosine	$C_{24}H_{47}NO_7$	12.1	442.3169	↓
	8	Palmitoyl glucuronide	$C_{22}H_{42}O_7$	9.1	463.2913	↑
	9	LysoPC (16:1 (9Z))	$C_{24}H_{48}NO_7P$	7.1	538.3150	↑
	10	LysoPC (18:2 (9Z,12Z))	$C_{26}H_{50}NO_7P$	7.5	564.3307	↑
	11	LysoPC (20:4 (8Z,11Z,14Z,17Z))	$C_{28}H_{48}NO_7P$	7.1	586.3150	↑
	12	LysoPC (20:4 (5Z,8Z,11Z,14Z))	$C_{28}H_{50}NO_7P$	7.5	588.3307	↑
	13	LysoPC (22:5 (4Z,7Z,10Z,13Z,16Z))	$C_{30}H_{52}NO_7P$	7.8	614.3463	↑

Supplementary Table S7

Potential biomarkers in response to SPF exposure.

Mode	No.	Compound	Formula	t_R (min)	Determined *m/z*	Trend
	1	L-Homocysteine	$C_4H_9NO_2S$	15.2	90.5069	↓
	2	Neurine	$C_5H_{13}NO$	0.8	104.107	↓
	3	5-Aminopentanoic acid	$C_5H_{11}NO_2$	0.8	118.0863	↓
	4	Phenylethylamine	$C_8H_{11}N$	14.8	122.0964	↑
	5	L-Histidinol	$C_6H_{11}N_3O$	12.2	124.0875	↓
	6	13,14-Dihydro-prostaglandin E1 (PGE_0)	$C_{20}H_{36}O_5$	13.8	190.1264	↑
	7	N-Acetyl-D-glucosamine	$C_8H_{15}NO_6$	10.8	204.0872	↑
	8	12-Oxo-20-trihydroxy-leukotriene B4	$C_{20}H_{30}O_7$	6.5	214.0888	↓
	9	7, 8-Dihydroneopterin	$C_9H_{13}N_5O_4$	13.8	238.0940	↑
	10	Hexadecasphinganine	$C_{16}H_{35}NO_2$	10.5	256.2635	↓
	11	Diisobutyl phthalate	$C_{16}H_{22}O_4$	8.5	279.1591	↓
	12	Sphingosine	$C_{18}H_{37}NO_2$	10.8	282.2797	↓
ESI$^+$	13	Sphinganine	$C_{18}H_{39}NO_2$	6.8	302.3054	↓
	14	Phytosphingosine	$C_{18}H_{39}NO_3$	6.2	318.3003	↓
	15	1-Monopalmitin	$C_{19}H_{38}O_4$	10.8	331.2843	↓
	16	Thromboxane A2 (TXA_2)	$C_{20}H_{40}O$	13.8	338.3417	↑
	17	Deoxycytidine monophosphate	$C_9H_{14}N_3O_7P$	10.8	371.0727	↑
	18	6,7-Dimethyl-8-(1-D-ribityl) lumazine	$C_{13}H_{18}N_4O_6$	12.2	409.183	↑
	19	Cholesteryl acetate	$C_{29}H_{48}O_2$	13.8	429.3727	↓
	20	Citicoline	$C_{14}H_{26}N_4O_{11}P_2$	13.8	471.1046	↑
	21	LysoPC (16:0)	$C_{24}H_{50}NO_7P$	8.2	496.3398	↓
	22	LysoPC (17:0)	$C_{25}H_{52}NO_7P$	8.8	510.3554	↓
	23	LysoPC (18:1 (9Z))	$C_{26}H_{52}NO_7P$	8.5	522.3554	↓
	24	LysoPC (18:0)	$C_{26}H_{54}NO_7P$	9.8	524.3711	↓
	25	LysoPC (20:3 (5Z,8Z,11Z))	$C_{28}H_{52}NO_7P$	7.8	546.3554	↓
	1	p-Cresol sulfate	$C_7H_8O_4S$	5.1	187.0071	↓
	2	D-Glucose	$C_6H_{12}O_6$	1.1	225.0616	↑
	3	Linoleic acid	$C_{18}H_{32}O_2$	10.8	279.233	↓
	4	Oleic acid	$C_{18}H_{34}O_2$	11.8	281.2486	↓
	5	Arachidonic acid	$C_{20}H_{32}O_2$	10.5	303.233	↓
ESI$^-$	6	20-Hydroxyeicosatetraenoic acid	$C_{20}H_{32}O_3$	8.1	319.2279	↓
	7	Docosahexaenoic acid	$C_{22}H_{32}O_2$	10.1	327.233	↓
	8	2-Methoxy-estradiol-17b 3-glucuronide	$C_{25}H_{34}O_9$	7.1	459.2019	↑
	9	LysoPC (15:0)	$C_{23}H_{48}NO_7P$	8.1	480.3096	↓
	10	LysoPC (18:2 (9Z,12Z))	$C_{26}H_{50}NO_7P$	7.5	564.3307	↓
	11	LysoPC (20:4 (5Z,8Z,11Z,14Z))	$C_{28}H_{50}NO_7P$	7.5	588.3307	↓

Supplementary Table S8

Effects of PF and SPF on the osteoporosis model ($\bar{x}$ ± s).

Groups	ALP (U/L)	wet bone coefficient of left femur (%)	dry bone coefficient of left femur (%)
Control	85.65 ± 3.70	0.26 ± 0.02	0.17 ± 0.02
OST model	75.22 ± 4.68**	0.22 ± 0.02*	0.13 ± 0.02**
ED	81.40 ± 2.09$^{\#}$	0.26 ± 0.03	0.16 ± 0.02$^{\#}$
PF	81.59 ± 2.11$^{\#}$	0.25 ± 0.02	0.15 ± 0.01$^{\#}$
SPF	85.18 ± 3.12$^{\#\#}$	0.27 ± 0.02$^{\#}$	0.18 ± 0.01$^{\#\#}$

$^{*}P < 0.05$, $^{**}P < 0.01$, compared with the control group; $^{\#}P < 0.05$, $^{\#\#}P < 0.01$, compared with the model group.

Supplementary Table S9

Effects of PF and SPF on the diarrhea model ($\bar{x}$ ± s).

Groups	Gastrin (ng/L)	Motilin (ng/L)
Control	207.62 ± 5.02	216.12 ± 4.34
DIA model	242.54 ± 4.63**	233.39 ± 4.68**
CD	224.30 ± 4.54$^{\#\#}$	214.71 ± 3.58$^{\#\#}$
PF	233.16 ± 4.07$^{\#}$	224.88 ± 6.27$^{\#}$
SPF	221.85 ± 4.92$^{\#\#}$	220.30 ± 5.55$^{\#\#}$

$^{**}P < 0.01$, compared with the control group; $^{\#}P < 0.05$, $^{\#\#}P < 0.01$, compared with the model group.

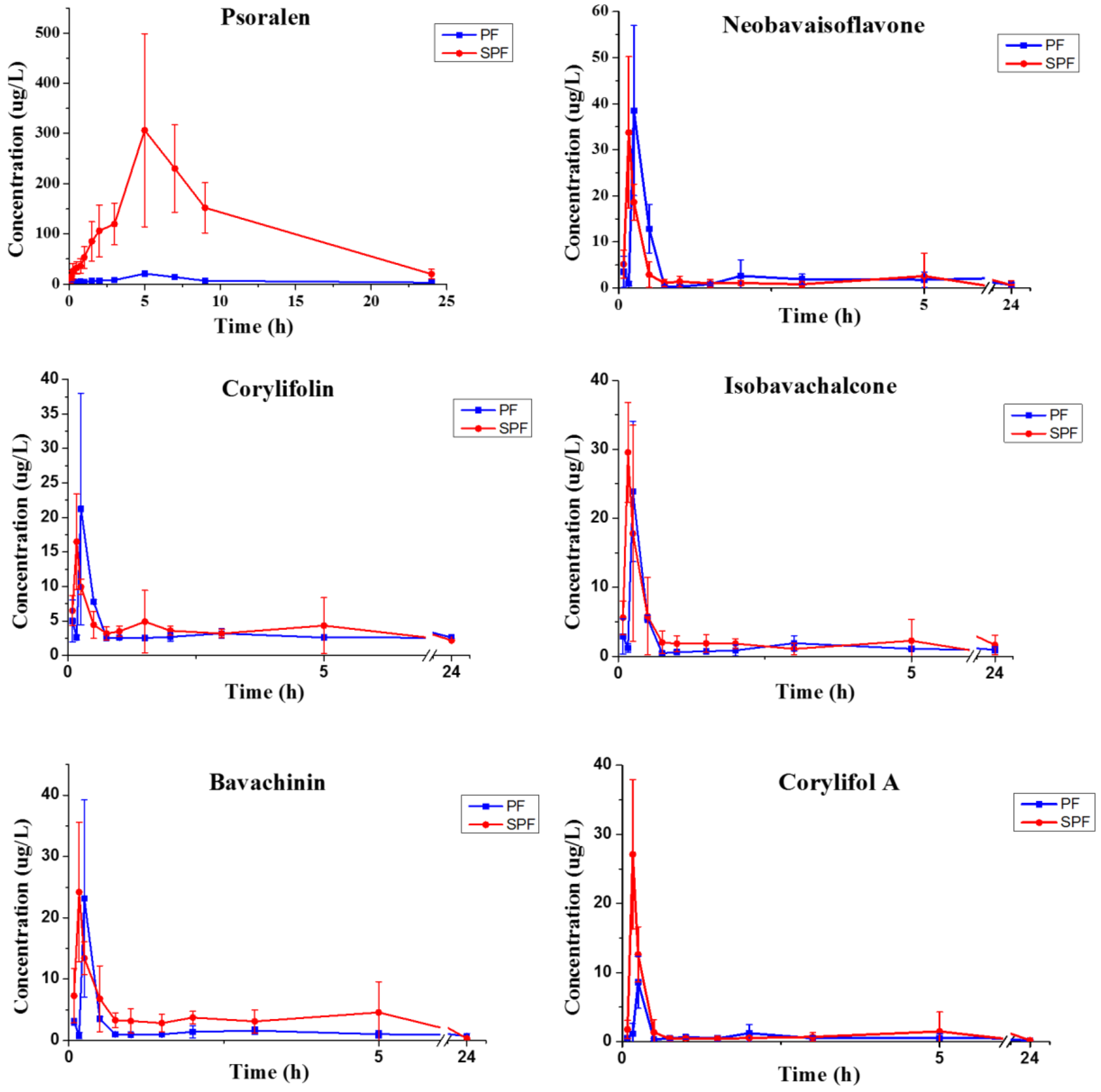

Supplementary Fig. S1. The plasma concentration-time curves of analytes after oral administration of PF and SPF extract (n = 6, mean ± SD).

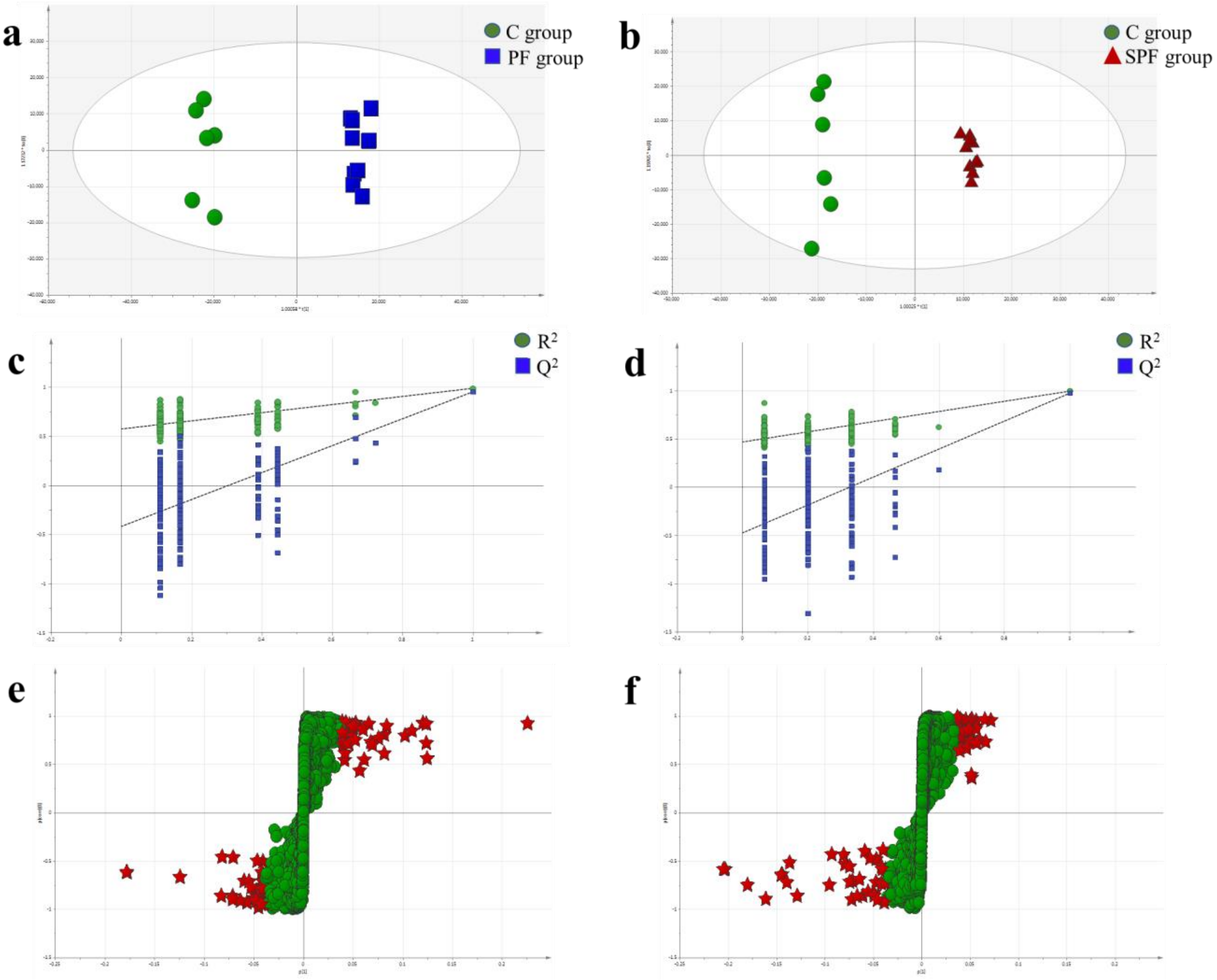

Supplementary Fig. S2. OPLS-DA score scatter plots, validation plots and S-plots obtained from control group and treatment group. OPLS-DA score scatter plots obtained from C *vs.* PF group (a), C *vs.* SPF group (b). Validation plots for OPLS-DA models obtained from C *vs.* PF group (c), C *vs.* SPF group (d). S-plots obtained from C *vs.* PF group (e), C *vs.* SPF group (f).

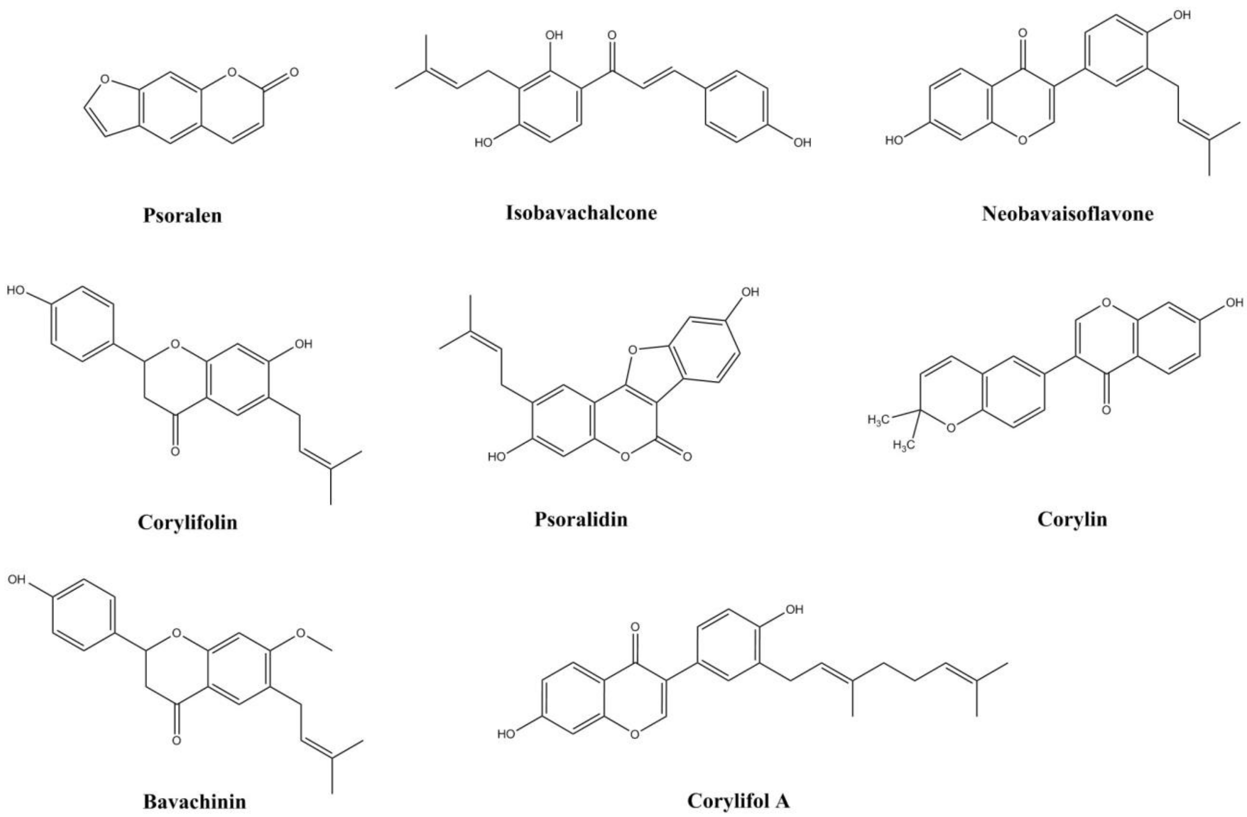

Supplementary Fig. S3. Chemical structures of psoralen, neobavaisoflavone, corylifolin, corylin, psoralidin, isobavachalcone, bavachinin and corylifol A.

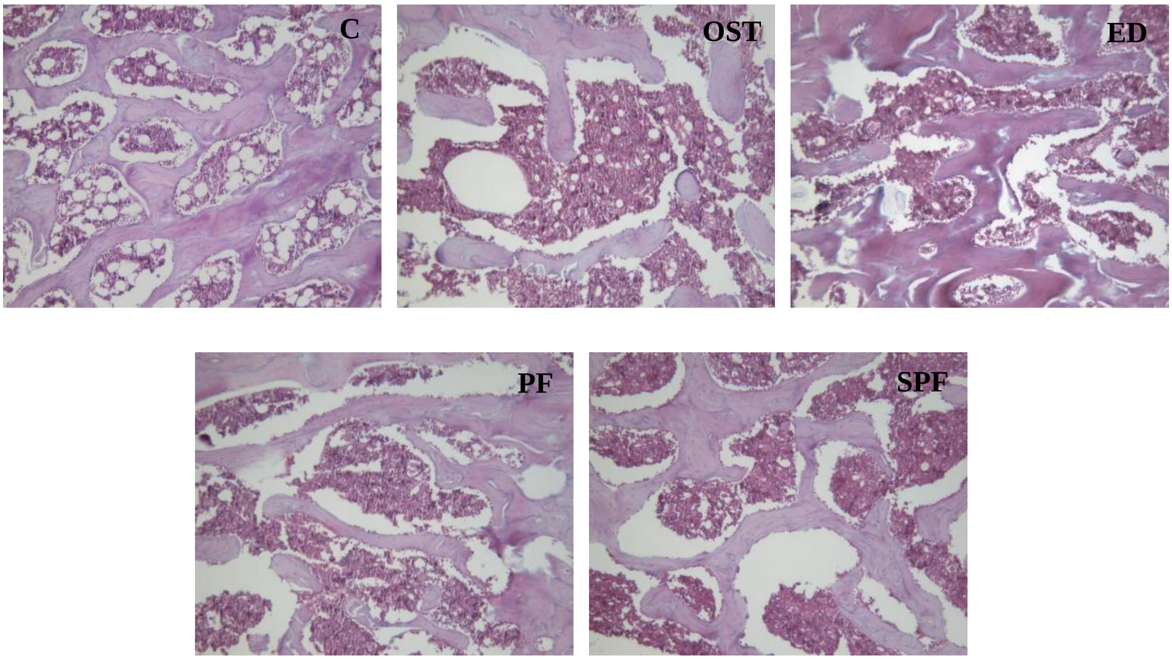

Supplementary Fig. S4. Histopathological examination (Magnification 40×) in C, OST model and treatment groups.
